# A comparative study of machine learning algorithms for predicting acute kidney injury after liver cancer resection

**DOI:** 10.7717/peerj.8583

**Published:** 2020-02-25

**Authors:** Lei Lei, Ying Wang, Qiong Xue, Jianhua Tong, Cheng-Mao Zhou, Jian-Jun Yang

**Affiliations:** Department of Anesthesiology, Pain and Perioperative Medicine, The First Affiliated Hospital of Zhengzhou University, Zhengzhou, China

**Keywords:** Machine learning, AKI, Hepatectomy, Postoperative, Secondary analysis

## Abstract

**Objective:**

Machine learning methods may have better or comparable predictive ability than traditional analysis. We explore machine learning methods to predict the likelihood of acute kidney injury after liver cancer resection.

**Methods:**

This is a secondary analysis cohort study. We reviewed data from patients who had undergone resection of primary hepatocellular carcinoma between January 2008 and October 2015.

**Results:**

The analysis included 1,173 hepatectomy patients, 77 (6.6%) of whom had AKI and 1,096 (93.4%) who did not. The importance matrix for the Gbdt algorithm model shows that age, cholesterol, tumor size, surgery duration and PLT were the five most important parameters. Figure 1 shows that Age, tumor size and surgery duration had weak positive correlations with AKI. Cholesterol and PLT also had weak negative correlations with AKI. The models constructed by the four machine learning algorithms in the training group were compared. Among the four machine learning algorithms, random forest and gbm had the highest accuracy, 0.989 and 0.970 respectively. The precision of four of the five algorithms was 1, random forest being the exception. Among the test group, gbm had the highest accuracy (0.932). Random forest and gbm had the highest precision, both being 0.333. The AUC values for the four algorithms were: Gbdt (0.772), gbm (0.725), forest (0.662) and DecisionTree (0.628).

**Conclusions:**

Machine learning technology can predict acute kidney injury after hepatectomy. Age, cholesterol, tumor size, surgery duration and PLT influence the likelihood and development of postoperative acute kidney injury.

## Introduction

Acute kidney injury (AKI) is a common postoperative complication among surgical patients. The incidence of postoperative AKI accounts for 18%–47% of total hospitalized AKI patients (Tang & Murray, 2004). Postoperative AKI can prolong the hospitalization period and increase the risk of both in-hospital mortality and chronic kidney disease. Clinically, postoperative AKI is easy to overlook, and the diagnostic rate is low (Moore et al., 2010; Bennet et al., 2010).

Hepatectomy is the most common aggressive treatment for primary liver cancer. In order to control hemorrhaging during surgery, it is often necessary to block the hepatic portal. This can disturb liver microcirculation. Hepatic ischemia-reperfusion injury occurs after the hepatic portal is opened, releasing a large amount of inflammatory media and oxygen free radicals, thus inhibiting liver function. At the same time, due to surgical trauma, decreased circulation in the liver and kidneys, the release of granulocyte elastase and other factors, postoperative renal damage is also common (Miranda et al., 2010). Therefore, although progress has been made on hepatectomy, the occurrence of AKI remains an important factor influencing prognosis (Nadeem et al., 2014).

**Figure 1:**
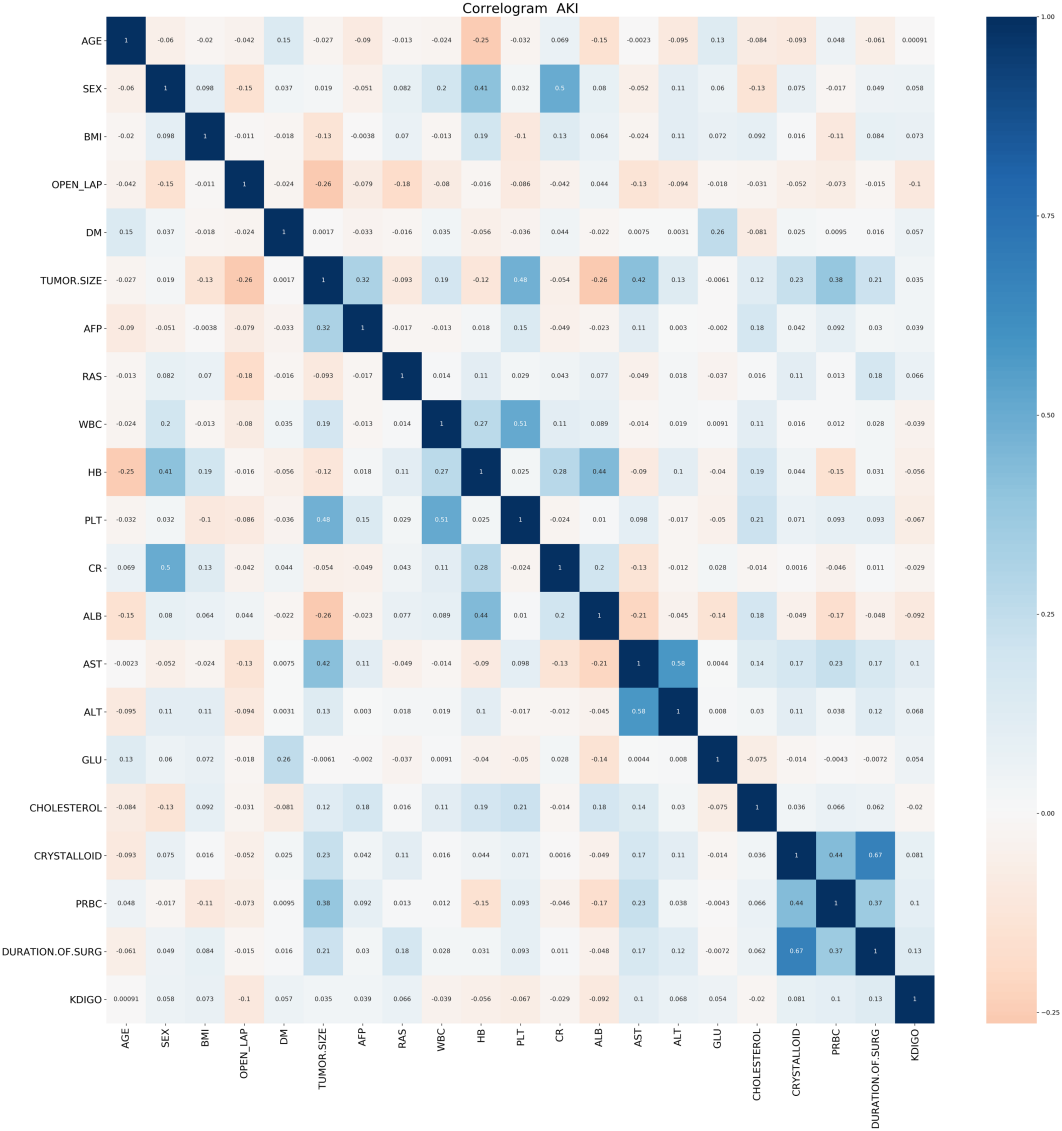
Correlation Analysis of various factors.

Many studies have used classical regression methods to identify risk factors and construct risk prediction models. However, a non-linear relationship between explanatory variables and outcome variables cannot be ruled out (Chen et al., 2011; Vives et al., 2016; Jun et al., 2018). However, compared with conventional analysis methods, machine learning techniques minimize these limitations and may perform better. Studies have shown that machine learning can predict AKI after liver transplant, cardiac surgery, severe burns and percutaneous coronary intervention (Lee et al., 2018a; Lee et al., 2018b; Tran et al., 2019; Huang et al., 2018). Other studies have shown that decision tree algorithms can predict hospitalized patients’ AKI risk after surgery (Thottakkara et al., 2016). Studies have also shown that support vector machines can be used as risk prediction models for postoperative AKI in septic patients (Mohamadlou et al., 2018) . This study investigated the preoperative risk factors associated with secondary AKI after hepatectomy. It used machine learning techniques (logistic regression, decision tree and GradientBoosting) to construct a predictive model of secondary AKI after hepatectomy, thus providing guidance for clinical therapies, and improving surgical patient prognosis.

## Materials and Methods

### Contributions of previous research

#### Study design

This is a secondary analysis cohort study. After this retrospective observational study was approved by the ethics committee of the Asan Medical Center, data for patients who had undergone primary hepatocellular carcinoma resection between January 2008 and October 2015 were reviewed. Since this research was retrospective, informed consent was waived. All surgical procedures were performed continuously by the same surgeon. Among the 1,184 identified patients, those with stage 3 or later serious chronic kidney disease (CKD) were excluded by a consulting nephrologist (*n* = 11). As serum creatinine level examination was part of the routine preoperative assessment, we referred patients with serum creatinine >1.5 mg/dL or patients with a history of CKD, to a consulting nephrologist for preoperative risk stratification. The final cohort included 1,173 patients.

#### Anesthesia and surgical technique

General anesthesia was performed with thiopental, fentanyl and rocuronium. Anesthesia was maintained with 2–4% sevoflurane in 50% air/oxygen. Routine invasive arterial blood pressure monitoring and central venous pressure monitoring were also conducted. Crystals and colloids were infused as well. The total hydroxyethyl starch volume did not exceed 20 mL/kg. When the patient’s hemoglobin was <8 mg/dL, red blood cells were infused. For patients with a history of ischemic heart disease, hemoglobin levels were maintained >10 mg/dL. Central venous pressure was maintained <5 mmHg. Vasoactive drugs were administered if the mean arterial blood pressure was <65 mmHg.

#### Indicator collection

The primary endpoint was AKI, based on the definition of the Kidney Disease: Improving Global Outcomes (KDIGO) Guidelines. Postoperative AKI was defined as an increase in serum creatinine ≥0.3 mg/dL within 2 days after surgery, or an increase ≥1.5-fold in serum creatinine within 7 days after surgery (Tran et al., 2019).

Patients’ baseline characteristics, laboratory variables and perioperative variables were collected. The baseline characteristics included age, sex, body mass index (BMI) and diabetes. Variables associated with tumor characteristics included, for example, alpha-fetoprotein. Laboratory data included hemoglobin, platelets, creatinine, white blood cell (WBC) count, glucose and total cholesterol. Intraoperative data included crysta and operative time.

### The methods were applied by the authors

The Python programming language (Python Software Foundation, version 3.6) was used for our analysis. The Scikit-learn package (Scikit Learning (https://github.com/scikit-learn/scikit-learn)) (Huang et al., 2018; Teles et al., 2016) was used for machine learning. This included forest, gbm, decision tree and Gbdt. The programming analysis code used in our research is shown in Appendix S1.

The sample was randomly divided into a training set and a test set, at a ratio of 7:3. The coefficients for the machine learning technique were trained with the training set and tested with the test set. Evaluation and comparison were completed with the prediction accuracy of a model constructed by machine learning and the area under the receiver operating characteristic curve. We also compared MSE, accuracy and recall rate. Missing data were estimated through multiple imputations.

F1-Measure evaluation indicators are often used in information retrieval and natural language processing. They constitute a comprehensive evaluation index based on precision rate and recall rate, and their specific definitions are as follows:

\begin{eqnarray*}& & F1=2rp/(r+p) \end{eqnarray*}

where *R* is the recall and *P* is the precision.

Precision rate indicates the proportion of correctly classified cases among the sample.

Accuracy rate indicates the number of paired cases divided by the total number of cases.

Recall rate indicates how many positive cases in the sample were predicted correctly.

#### Machine learning algorithm

In machine learning, a random forest (forest) is a classifier that includes multiple decision trees. The categories of its output are determined by the modes of categories output by individual trees.

The LightGBM (gbm) algorithm is a lifting machine learning algorithm. It is a fast, distributed and high-performing gradient lifting framework based on a decision tree algorithm. It can sort, classify, run regressions, and perform many other machine learning tasks.

The construction of a decision tree model has two steps: induction and pruning. Induction is the step of constructing a decision tree (tr) by setting all hierarchical decision boundaries based on data at hand. However, the tree model is subject to severe over-fitting due to the nature of the training decision tree, and this is when pruning is required. Pruning is the process of removing unnecessary branch structures from the decision tree, simplifying the process of overcoming over-fitting and making it easier to interpret.

Elevation is a machine learning technique that can be used for regression and classification problems. It produces a weak prediction model (like a decision tree) at each step and weights it into the total model. If the weak prediction model of each step generates consistent loss function gradient direction, then it is called gradient boosting (Gbdt).

## Results

The pandas_profiling package was applied to data exploration (see attachment Appendix S1 for the results) with Python. The analysis included 1,173 hepatectomy patients, including 77 patients (6.6%) with AKI and 1,096 (93.4%) without. The BMI values of the two groups were different, and the difference was statistically significant (*P* < 0.040). Neither age nor tumor size showed statistically significant difference between the two groups (see Table 1).

**Figure 2:**
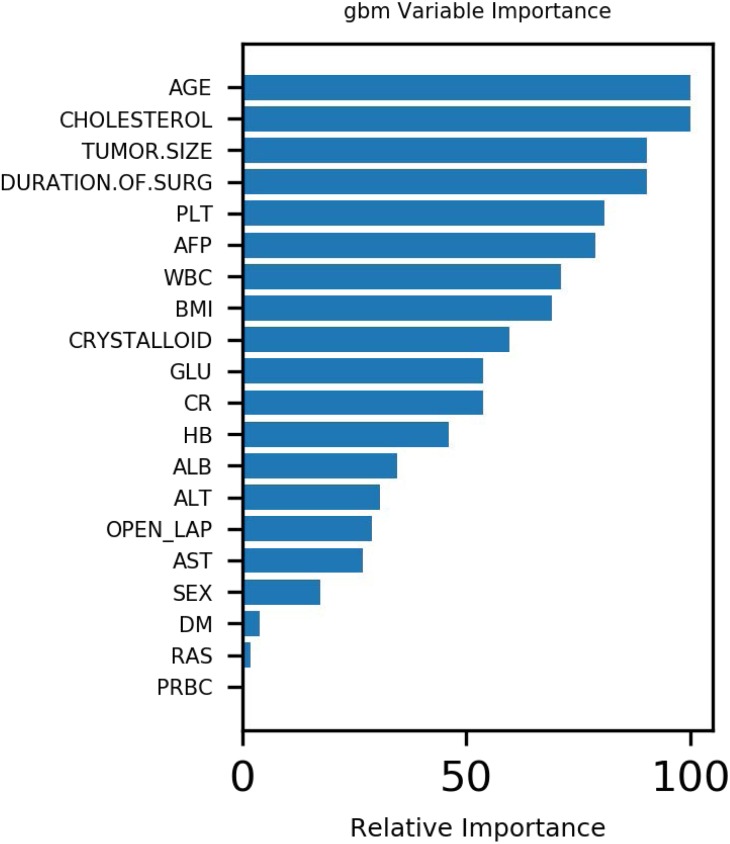
Variable importance of features included in Gbdt algorithm for prediction of AKI.

Figure 1 demonstrates that age, tumor size and surgery duration have weak positive correlations with AKI. Cholesterol and PLT each had weak negative correlations with AKI.The Gbdt algorithm model importance matrix is shown in Fig. 2. Age, cholesterol, tumor size, surgery duration and PLT are the five most influential factors.

**Figure 3:**
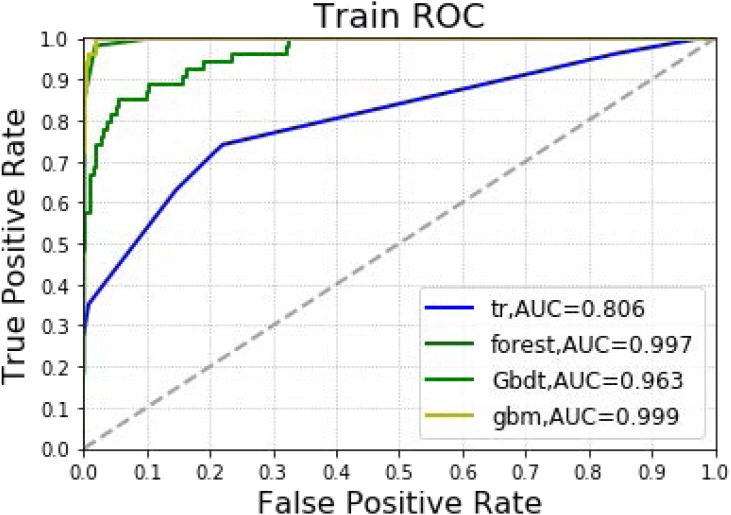
Machine learning algorithm for prediction of AKI in training group.

In Table 2 and Fig. 3, the models constructed by the four machine learning algorithms in the training group are compared. Among the four machine learning algorithms, random forest and gbm have the highest accuracy, 0.989 and 0.970 respectively. The precision of four of the five algorithms is 1, with random forest as the lone exception. The highest recall rate was that of the random forest algorithm (0.852). Among the four algorithms, random forest had the highest recall rate and f1 score, 0.852 and 0.911, respectively. The AUC values for the four algorithms were: gbm (0.999), forest (0.997), Gbdt (0.963) and DecisionTree (0.806). Among the four algorithms, random forest had the lowest MSE value (0.011).

**Figure 4:**
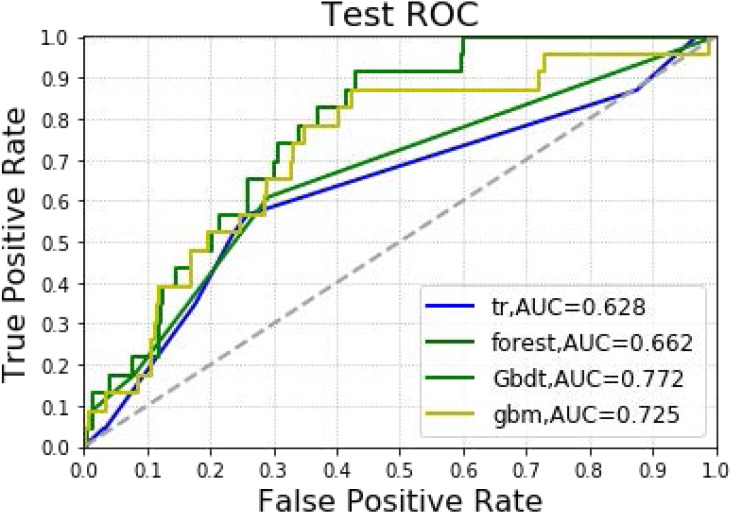
Machine learning algorithm for prediction of AKI in the testing group.

In Table 3 and Fig. 4, the models constructed by four machine learning algorithms in the test group are compared. Gbm had the highest accuracy (0.932). Random forest and gbm had the highest precision, both being 0.333. The recall rate for the random forest algorithm was 0.087. The lowest f1 score was that of decision tree at 0.059. The AUC values of the four algorithms were: Gbdt (0.772), gbm (0.725), forest (0.662) and DecisionTree (0.628). Among the four algorithms, gbm had the lowest MSE value at 0.068.

## Discussion

Hepatectomy is an effective therapy for primary liver cancer. To block interoperative bleeding, it is often necessary to block the hepatic hilum. This can induce hepatic ischemia-reperfusion injury. It can cause not only liver dysfunction, but also kidney injury (Sheridan et al., 2016; Gao et al., 2018). At the same time, due to surgical trauma, decreased blood flow in the liver and decreased kidney circulation, granulocyte elastase release and other factors, postoperative renal damage can also occur (Fonseca-NetoI et al., 2012). In this study, machine learning techniques compared the predictive accuracy of AKI predictions after hepatectomy. The Gbdt algorithm indicated that age, cholesterol, tumor size, surgery duration and PLT were the five most important weights for AKI. The results showed that Gbdt had the highest AUC in both training and test groups. Thus, it could predict the likelihood of AKI. All four machine learning algorithms could predict the likelihood of AKI as well. The accuracy was greater than 90%, and the MSE values were less than 0.1.

**Table 1:** Clinical basic characteristic information.

AKI	NO	Yes	*P*-value
*N*	1,096	77	
AGE (years)	55.7 ± 10.3	55.7 ± 9.3	0.789
BMI (kg/m^2^)	24.2 ± 2.8	25.0 ± 3.2	0.040
TUMOR SIZE (cm)	4.5 ± 3.7	5.1 ± 4.2	0.510
AFP	9057.7 ± 59451.3	18930.6 ± 105276.9	0.046
WBC (×10^3^/µL)	5.4 ± 1.8	5.2 ± 1.5	0.365
HB (mg/dL)	14.0 ± 1.6	13.6 ± 1.6	0.059
PLT (×10^3^/µL)	165.1 ± 66.5	147.2 ± 68.1	0.002
CR (mg/dL)	0.8 ± 0.2	0.8 ± 0.2	0.135
ALB (g/dL)	3.8 ± 0.4	3.7 ± 0.4	0.008
AST (IU/L)	39.0 ± 28.9	51.6 ± 47.6	0.002
ALT (IU/L)	36.6 ± 27.8	44.2 ± 31.5	0.010
GLU (mg/dL)	117.8 ± 45.8	128.1 ± 63.1	0.626
CHOLESTEROL (mg/dL)	163.7 ± 34.6	160.8 ± 43.3	0.138
PRBC (units)	0.2 ± 1.0	0.6 ± 2.4	0.001
CRYSTALLOID (mL)	2242.5 ± 934.7	2562.5 ± 1491.9	0.140
Duration of surgery (min)	268.2 ± 79.5	311.9 ± 93.9	<0.001
SEX			0.048
Female	214 (19.5%)	8 (10.4%)	
Male	882 (80.5%)	69 (89.6%)	
OPEN_LAP			<0.001
No	853 (77.8%)	73 (94.8%)	
Yes	243 (22.2%)	4 (5.2%)	
DM			0.085
No	1,030 (94.0%)	68 (88.3%)	
Yes	66 (6.0%)	9 (11.7%)	
RAS			0.023
No	932 (85.0%)	58 (75.3%)	
Yes	164 (15.0%)	19 (24.7%)	

**Notes.**

WBC white blood cell HB Hemoglobin DM Diabetes BMI Body index CR Creatinine GLU Glucose RAS Renin-angiotensin system (RAS) blocker

Studies (Craig et al., 2001; Yim et al., 2000; Amar et al., 2007) have shown that laparoscopic surgery can reduce postoperative inflammatory response indicator levels, including C-reactive protein, interleukin and reactive oxygen species in neutrophils. These inflammatory mediators have been shown to be identical to the inflammatory mediators in AKI (Wu et al., 2014). In addition, triglyceride deposition around the renal tubules can cause high levels of free fatty acids around the kidney cells. This can impair kidney function (Levine et al., 1997). Zhang et al. (2011) analyzed 3,336 patients from 19 related studies covering 11 countries and found that blood CysC is a good predictor of acute kidney injury. It also has high specificity and accuracy for early kidney injury. These findings are similar to those of the present study.

Ongoing studies (Slankamenac et al., 2009) also show that diabetes, high BMI and low postoperative albumin are risk factors for postoperative AKI. Diabetes is a well-known risk factor for various postoperative AKIs, including hepatectomy. Low serum albumin concentrations have recently been associated with various postoperative AKIs (Cho et al., 2014). Moreover, studies (Wu et al., 2020) have also shown that the lowest platelet count over the first 48 h is a new biomarker for AKI. This study’s findings support these views.

The goal of logistic regression in statistics is different from that of logistic regression in machine learning. By default, there is a potential law in statistics. There are various restrictions in adjusting the model to meet the assumption conditions to find the potential law. However, machine learning is different; it is only concerned with the deviation between predicted and real values. Moreover, the integration algorithm adopted in this study considers more information gain when calculating. Thus, it naturally eliminates linear correlation, and also prevents non-linear correlation.

In addition, variables are often screened with principal component analysis (Zhang & Castelló, 2017). However, principal component analysis is not always required in machine learning algorithms. It is used excessively to screen features. Doing so can omit important factors for outcome variables. In the real world, no clinical factor affecting prognosis should be ignored.

**Table 2:** Forecast results of training group.

	Accuracy	Precision	Recall	f1_score	Auc	MSE
Decision Tree	0.952	1.000	0.278	0.435	0.806	0.048
forest	0.989	0.979	0.852	0.911	0.997	0.011
Gbdt	0.946	1.000	0.185	0.312	0.963	0.054
gbm	0.970	1.000	0.537	0.699	0.999	0.030

**Table 3:** Forecast results of testing group.

	Accuracy	Precision	Recall	f1_score	Auc	MSE
Decision Tree	0.909	0.091	0.043	0.059	0.628	0.091
forest	0.929	0.333	0.087	0.138	0.662	0.071
Gbdt	0.929	0.250	0.043	0.074	0.772	0.071
gbm	0.932	0.333	0.043	0.077	0.725	0.068

This study has several limitations. Firstly, not all confounding factors could be controlled, as this was a retrospective study. Secondly, caution should be exercised in interpreting the study’s results since it was a single-center study in which all surgeries were performed by an experienced surgeon. Thirdly, the machine learning techniques’ performance may vary when applied to larger samples with different covariate distributions. This study only performed internal, and not external, verification. In addition, different parameters in machine learning can result in different AUC values. Corresponding models are needed for different occasions according to needs, and should not excessively prioritize AUC values. Furthermore, an exorbitant AUC value may be unsuitable, as the accuracy, precision and recall rates may fall to unacceptable levels. This would make models unreliable in real world applications when the AUC value is high. Although most of the important reported variables are not clinically modifiable, appropriate measures could be taken to personalize prevention based on AKI risk.

## Conclusion

This study shows that all four machine learning techniques can predict AKI likelihood, among which GradientBoosting performs the best. At the same time, the Gbdt algorithm suggests that age, cholesterol, tumor size, surgery duration and PLT are the five most important weights for the likelihood of acute kidney injury after liver cancer resection.

##  Supplemental Information

10.7717/peerj.8583/supp-1 Appendix S1 Appendix Code 1

10.7717/peerj.8583/supp-2 Data S1 Raw data

## Additional Information and Declarations

### Data Availability

The following information was supplied regarding data availability: The programming analysis code is available as Supplemental File. Data is available at BioStudies database: S-EPMC5640237.
